# Supplementary material for: ZFP36-family RNA-binding proteins in regulatory T cells reinforce immune homeostasis
Source: Nat Commun. 2025 May 6;16:4192. doi: 10.1038/s41467-025-58993-y (PMC12056042; doi:10.1038/s41467-025-58993-y)
Supplement: Supplementary file 1 — Supplementary Information [file 41467_2025_58993_MOESM1_ESM.pdf]

Supplementary Figure 1

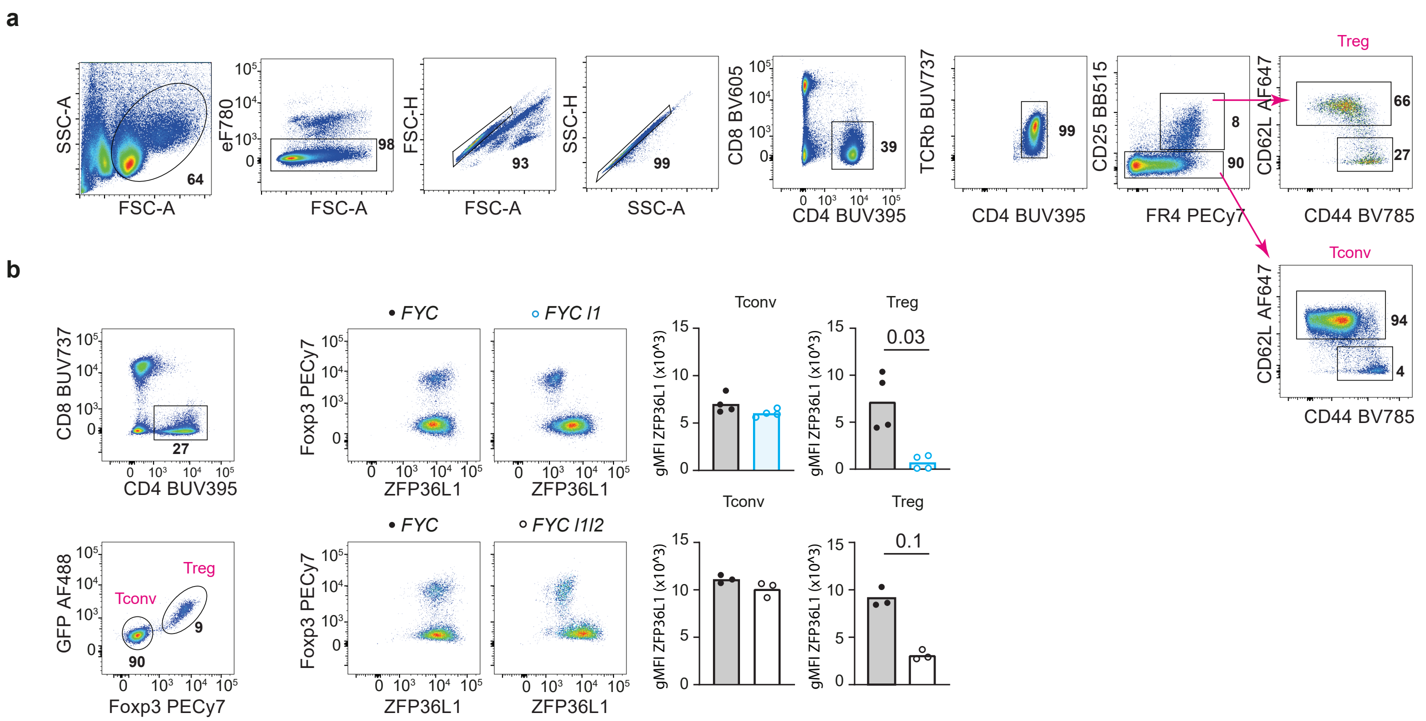

Supplementary Fig. 1 Phenotyping of T cell subsets

a, Gating strategy for data shown in Fig. 1a; Treg: $CD4^+FR4^+CD25^+$; and Tconv: $CD4^+CD25^-$ T cells.

b, Gating strategy and representative FACS plots comparing expression of ZFP36L1 in Treg ($CD4^+$ $FOXP3^+$ YFP^+) or Tconv ($CD4^+$ $FOXP3^-$ YFP^-) cells from *FYC, FYC l1* (upper panel) and *FYCl1l2* mice (lower panel) after stimulation with PMA and ionomycin for four hours at 37°C; cells were pre-gated on live, single cells; quantification of ZFP36L1 gMFI; key as shown.

Supplementary Figure 2

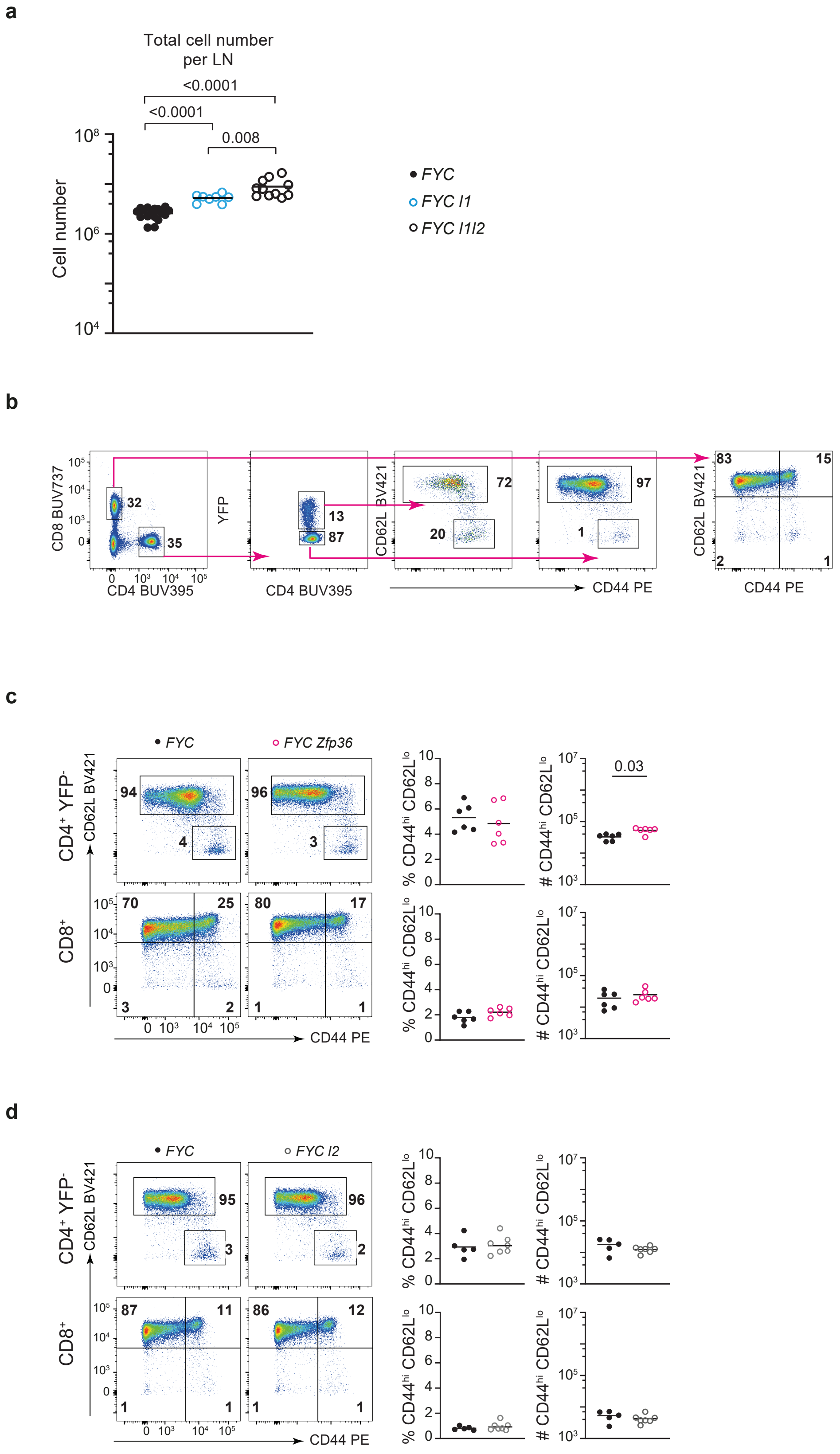

Supplementary Fig. 2 Characterisation of T cell subsets

a, Total cell number per LN; Control *icre*-only *FYC* (n=17); *FYC l1* (n=8)*; FYC l1l2* (n=11); key as shown.

Values are for LN cells from apparently healthy mice aged 10 -14 weeks. Data are from at least two independent experiments.

b, Gating strategy for CD4 and CD8 data shown in **Fig. 1d,e,f**.

c, **d**, Representative FACS plots and proportion and cell number of effector cells $CD44^{hi}CD62L^{lo}$ in the $CD4^{+}$ YFP^{-} (upper panel) and $CD8^{+}$ subsets (lower panel) from *FYC Zfp36* (**c**), and *FYC Zfp36l2* mice (**d**), analyzed with contemporaneous *FYC* controls. *FYC* (n=5-6); *FYC Zfp36* (n=6); *FYC Zfp36l2* (n=7); key as shown Numbers are shown per single LN in mice aged 9 -16 weeks. P values were determined using one-way ANOVA using multiple comparison (a) or Mann-Whitney test (c, d), or not shown where $p>0.05$.

Supplementary Figure 3

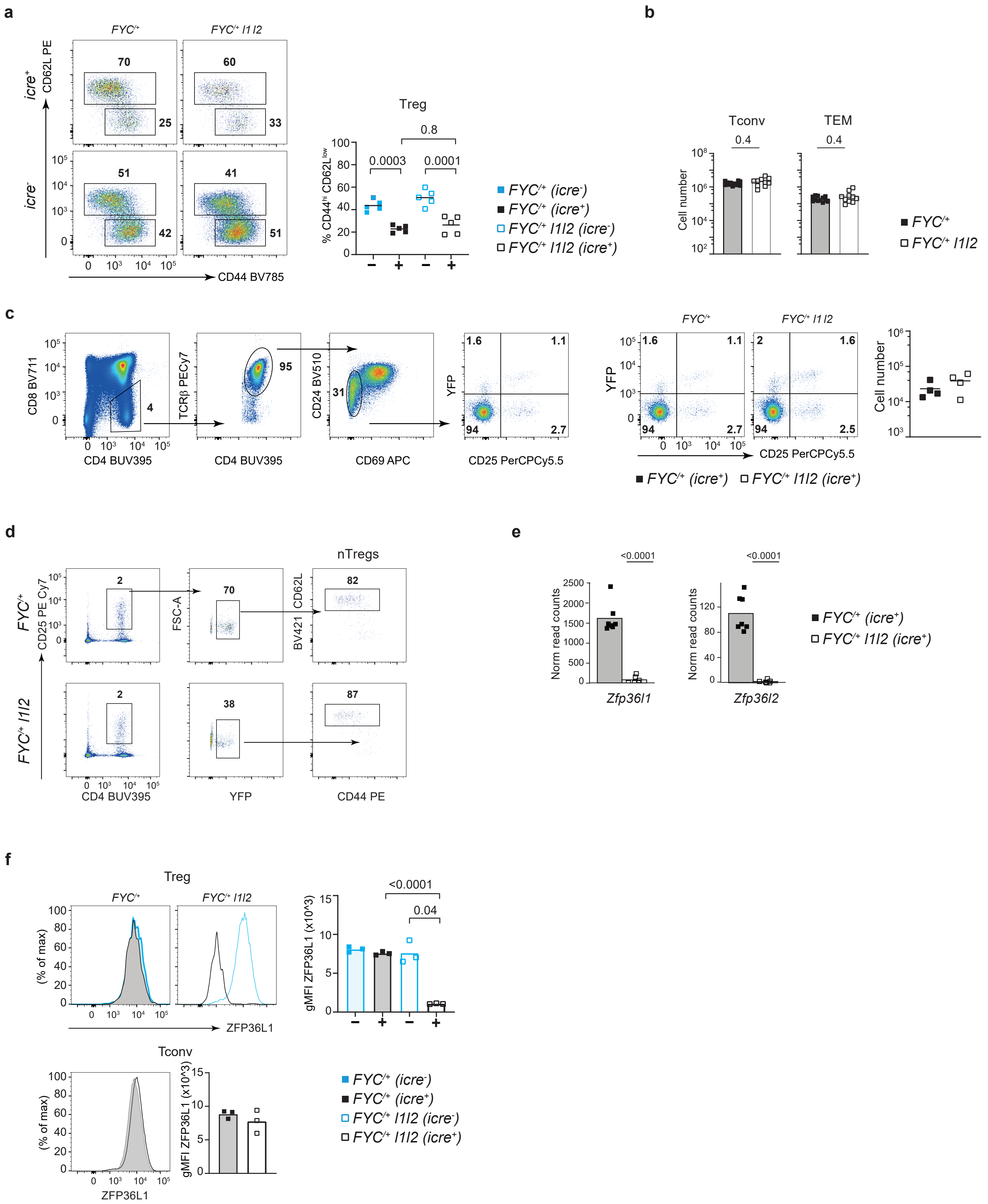

Supplementary Fig. 3 Compromised fitness of RBP-deficient Treg

a, Representative FACS plots and percentage of eTreg in the LN from FYC/+ and FYC/+ I1I2 mice; key as shown.

b, Number of effector cells in the Tconv (CD44^{hi}CD62L^{lo}) and CD8^{+} TEM (CD44^{hi}CD62L^{lo}) subsets.

c, Gating strategy and representative FACS plots comparing CD4^{+}TCRβ^{+}CD69^{-}YFP^{+} CD25^{+} Treg in the thymus of seven-week-old *FYC*$^{/+}$ and *FYC*$^{/+}$ *I1I2* mice; n=4

d, Gating strategy for sorting nTreg.

e, Normalised read counts across the loxP-flanked regions of the conditional *Zfp36l1* and *Zfp36l2* alleles; n=6-7; key as shown. Read counts were normalised using size factors derived from the overall DESeq2 analysis of all genes; p values determined using t test with FDR correction.

f, Representative histogram overlays comparing expression of ZFP36L1 in Treg (CD4^{+} FOXP3^{+}) cells from *FYC*$^{/+}$ and *FYC*$^{/+}$ *I1I2* mice (*icre*-positive (FOXP^{+}YFP^{+}) cells - black line and black symbols; *icre*-negative FOXP^{+}YFP^{-}) cells – blue line and blue symbols); or Tconv (CD4^{+} FOXP3^{-}) cells (lower panel). Cells were stimulated with PMA and ionomycin for four hours; plots showing gMFI for ZFP36L1 staining; n=3; key as shown.

P values determined using one-way ANOVA with multiple comparison (a, f) or Mann-Whitney (b, e).

Supplementary Figure 4

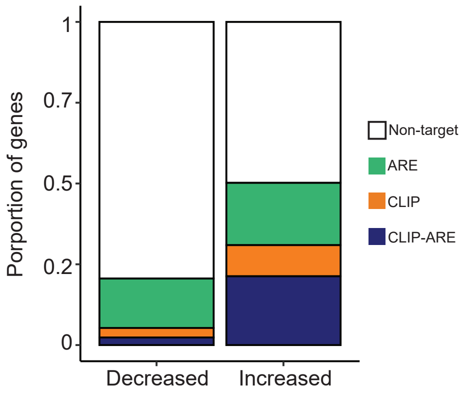

Supplementary Fig. 4 DE genes classified as ARE, CLIP or CLIP-ARE

Proportion of genes with changes in expression in the RNAseq dataset from *FYC/*$^{+}$ *I1I2* Treg and *FYC/*$^{+}$ Treg according to the presence of an ARE (defined as 2 x UAUU separated by up to 3 nucleotides) within their 3'UTR (green), ZFP36-family binding detected in their 3'UTR by CLIP (orange), or CLIP targets containing an ARE in their 3'UTR (dark blue) compared with non-target genes (grey).

Supplementary Figure 5

a

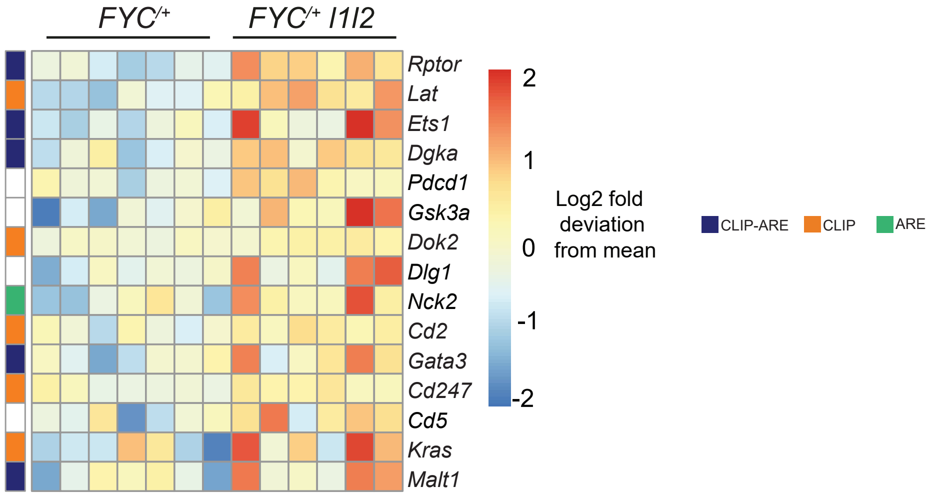

b

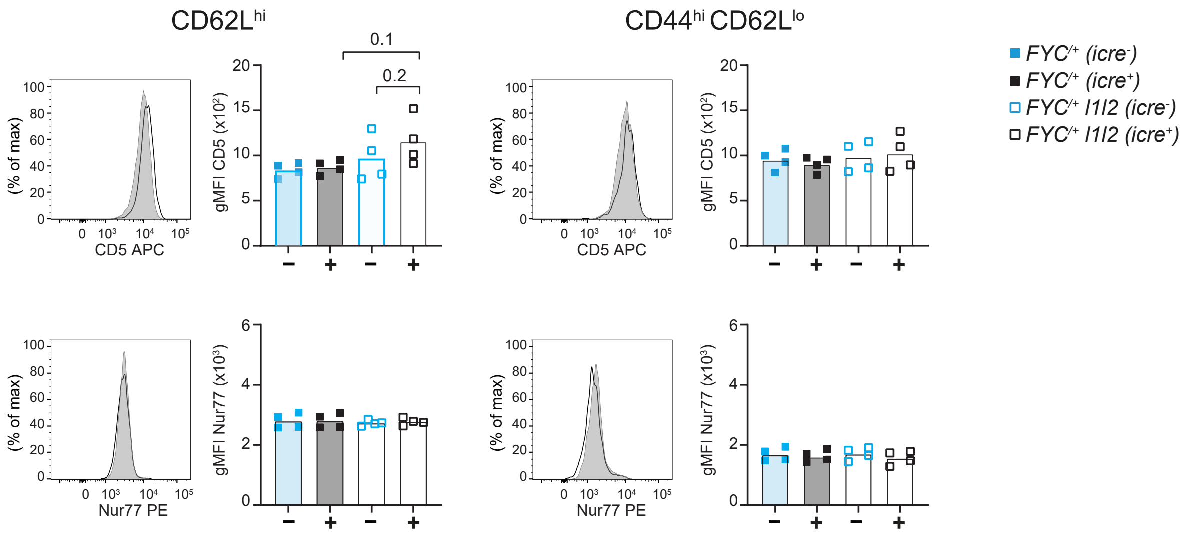

Supplementary Fig. 5 Genes enriched in the TCR signaling pathway

a, The heatmap depicts the top 15 ranked genes in the TCR signaling pathway, ordered with the most significantly increased genes at the top. The color scale represents the $\log_2$ fold deviation from the mean for each gene. Key for genes containing ARE, CLIP or CLIP-ARE as shown.

b, CD5 (upper panel) and NUR77 (lower panel) expression (gMFI) in $CD4^+$ $FOXP3^+$ nTreg ($CD62L^{hi}$) and eTreg ($CD44^{hi}$ $CD62L^{lo}$) from control $FYC^{/+}$ and $FYC^{/+}$ *I1I2* mice. n=4; key as shown.

P values in (b) determined using one-way ANOVA with multiple comparison.

Supplementary Figure 6

a

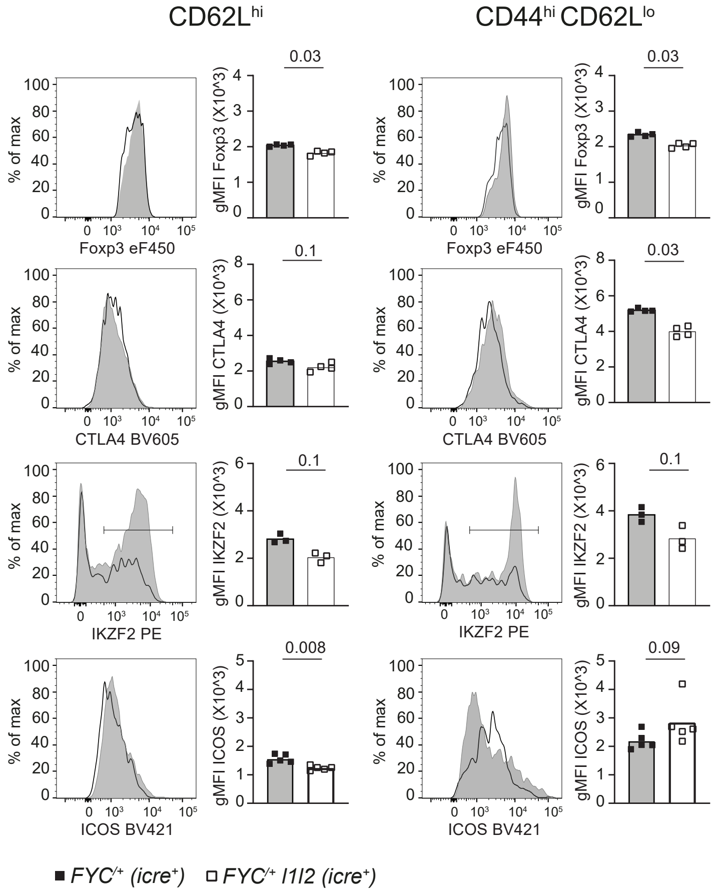

b

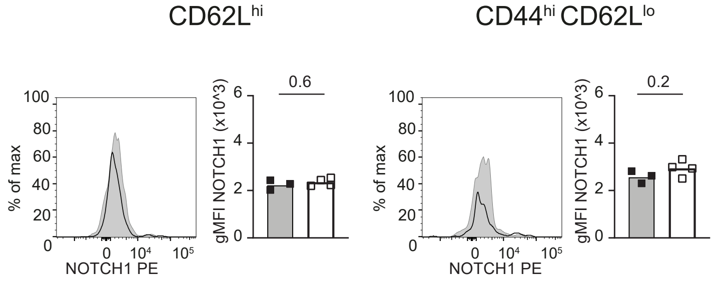

Supplementary Fig. 6 Expression of Treg signature genes

a, Representative FACS plots showing FOXP3, CTLA-4 (from LN, fixed *ex vivo*), IKZF2 (from mLN) and ICOS (from LN) expression (gMFI) in $CD4^{+}$ $FOXP3^{+}$ nTreg and eTreg from control *FYC*$^{/+}$ and *FYC*$^{/+}$*I1I2* mice; n=3-5; key as shown.

b, NOTCH1 expression (gMFI) in nTreg and eTreg from *FYC*$^{/+}$ and *FYC*$^{/+}$*I1I2* mice; n=3-4, key as in a. P values determined using Mann-Whitney.

Supplementary Figure 7

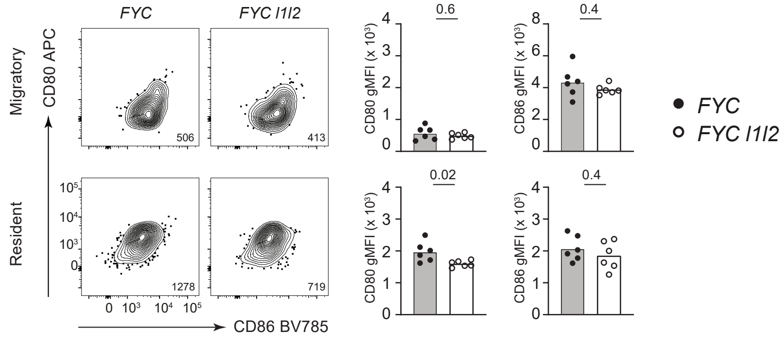

Supplementary Fig. 7 CD80 and CD86 expression in DC1

Representative FACS plots showing CD80 and CD86 expression on CD11c^{int} MHCII^{hi} “migratory” (upper panel) and CD11c^{hi} MHCII^{int} “resident” (lower panel) CD172a^{-} XCR1^{+} cDC1 from spleen from *FYC I1I2 and FYC* male mice; the number of events in the file is indicated; n=6, key as shown.

Supplementary Figure 8

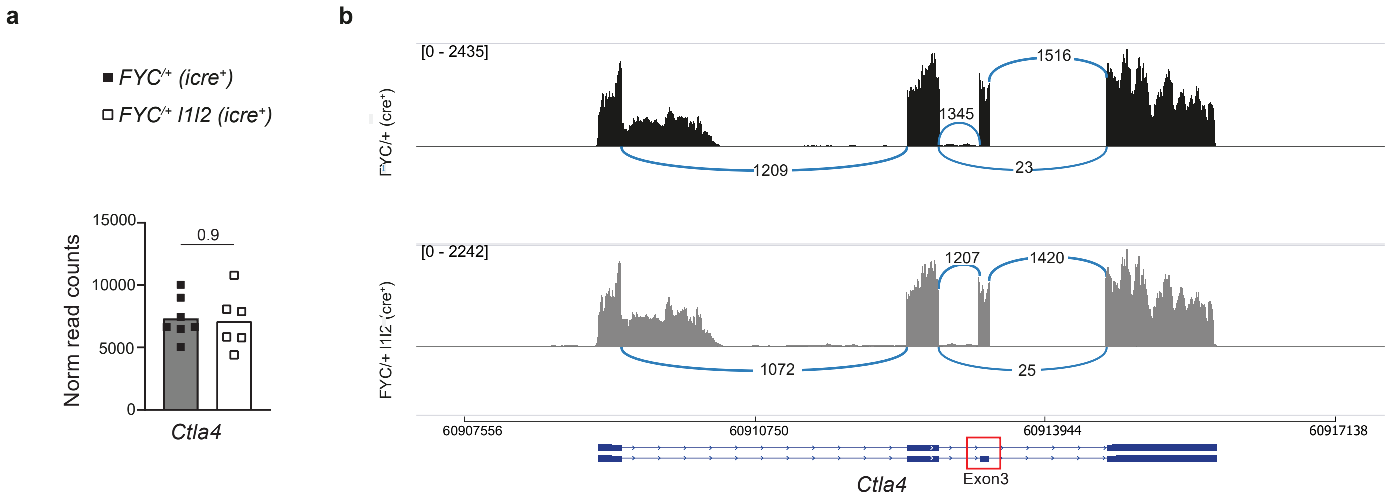

Supplementary Fig. 8 *Ctla4* transcript expression

a, *Ctla4* normalised read counts in YFP^{+} nTreg from *FYC*$^{/+}$ and *FYC*$^{/+}$ *I1I2* mice; n=6-7; p values determined using t test with FDR correction.

b, *Ctla4* transcript isoforms showing read coverage and reads that map to exon–exon junctions (displayed in a Sashimi plot; arches: numbers indicate reads that map to that junction). Exon 3 encoding the transmembrane domain is indicated by a red box.

Supplementary Figure 9

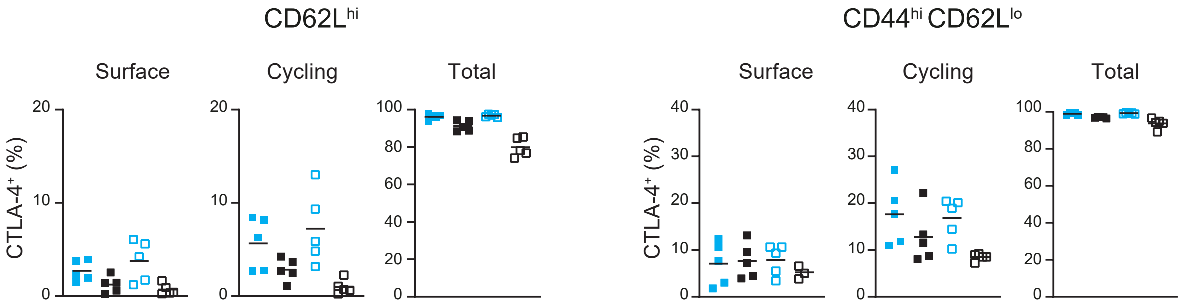

CD62L hi, Surface:

	■	□	□
■	0.04	0.9	0.002
■		0.01	0.4
□			0.0009

CD62L hi, Cycling:

	■	□	□
■	0.06	0.7	<0.0001
■		0.008	0.0008
□			<0.0001

CD62L hi, Total:

	■	□	□
■	0.05	0.9	<0.0001
■		<0.0001	<0.0001
□			<0.0001

CD44 hi CD62L lo, Surface:

	■	□	□
■	0.6	0.5	0.9
■		0.9	0.4
□			0.2

CD44 hi CD62L lo, Cycling:

	■	□	□
■	0.2	0.9	0.003
■		0.2	0.1
□			0.004

CD44 hi CD62L lo, Total:

	■	□	□
■	0.2	0.9	0.0003
■		0.1	0.01
□			0.0002

■ *FYC/+ (icre-)* ■ *FYC/+ (icre+)* □ *FYC/+ I1I2 (icre-)* □ *FYC/+ I1I2 (icre+)*

Supplementary Fig. 9 Decreased cycling of CTLA-4 in RBP-deficient nTreg

Percentage of CTLA-4^{+} nTreg or eTreg in surface, cycling, or total pool from *FYC*$^{/+}$ *I1I2 and FYC*$^{/+}$ female mice. Each symbol represents an individual mouse (n=5); key as shown. Data from at least two independent experiments.

P values determined using one-way ANOVA with multiple comparison are represented in the table shown.

Supplementary Figure 10

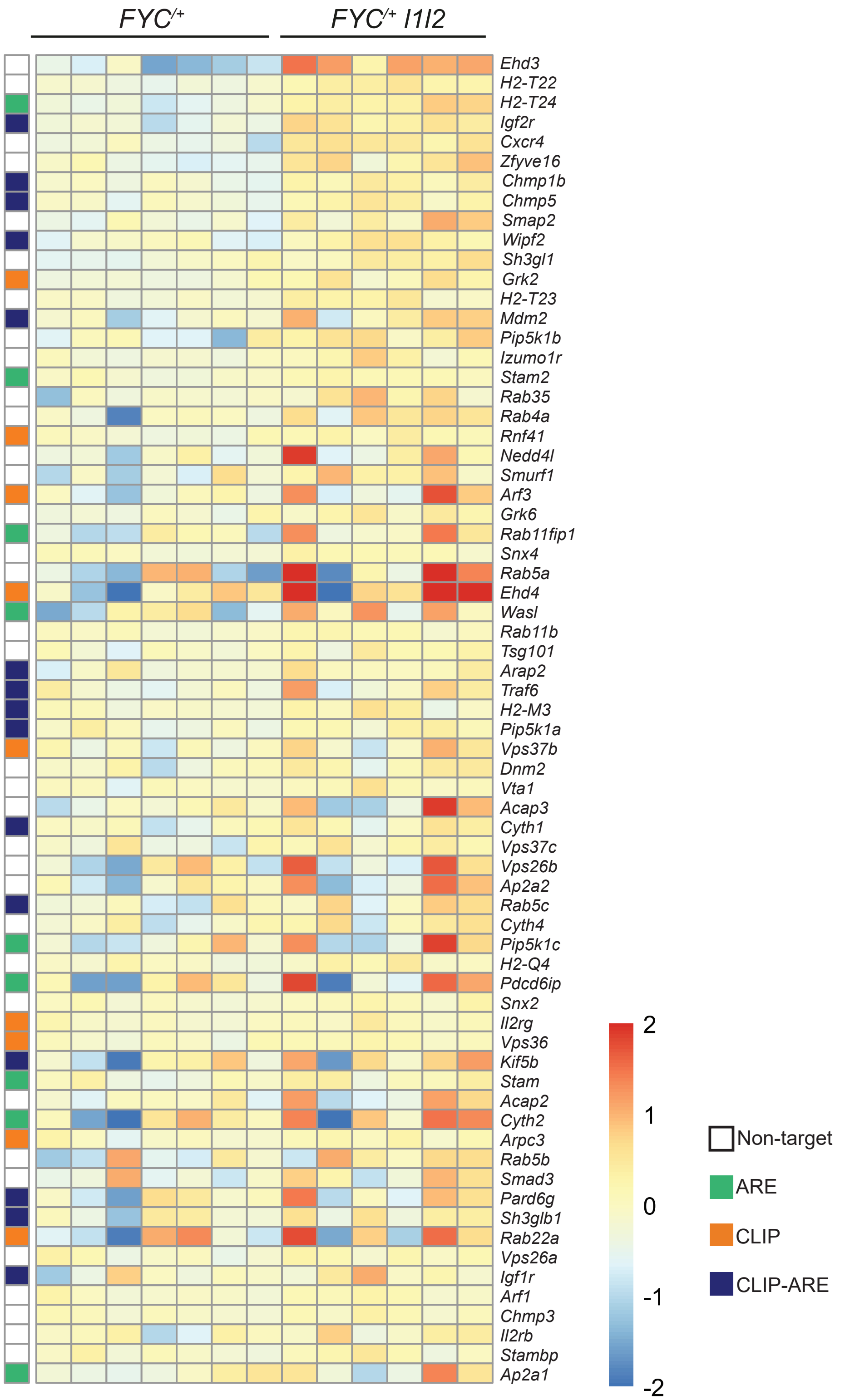

Supplementary Fig. 10 DE genes involved in endocytosis

a, Heatmap depicting the genes in the leading edge from GSEA analysis of the endocytosis pathway, ordered by FDR (with the most significantly increased genes at the top); genes with average normalised read count > 100 are included. The color scale represents the log2 fold deviation from the mean for each gene. Key for genes containing ARE, CLIP or CLIP-ARE as shown.

Supplementary Figure 11

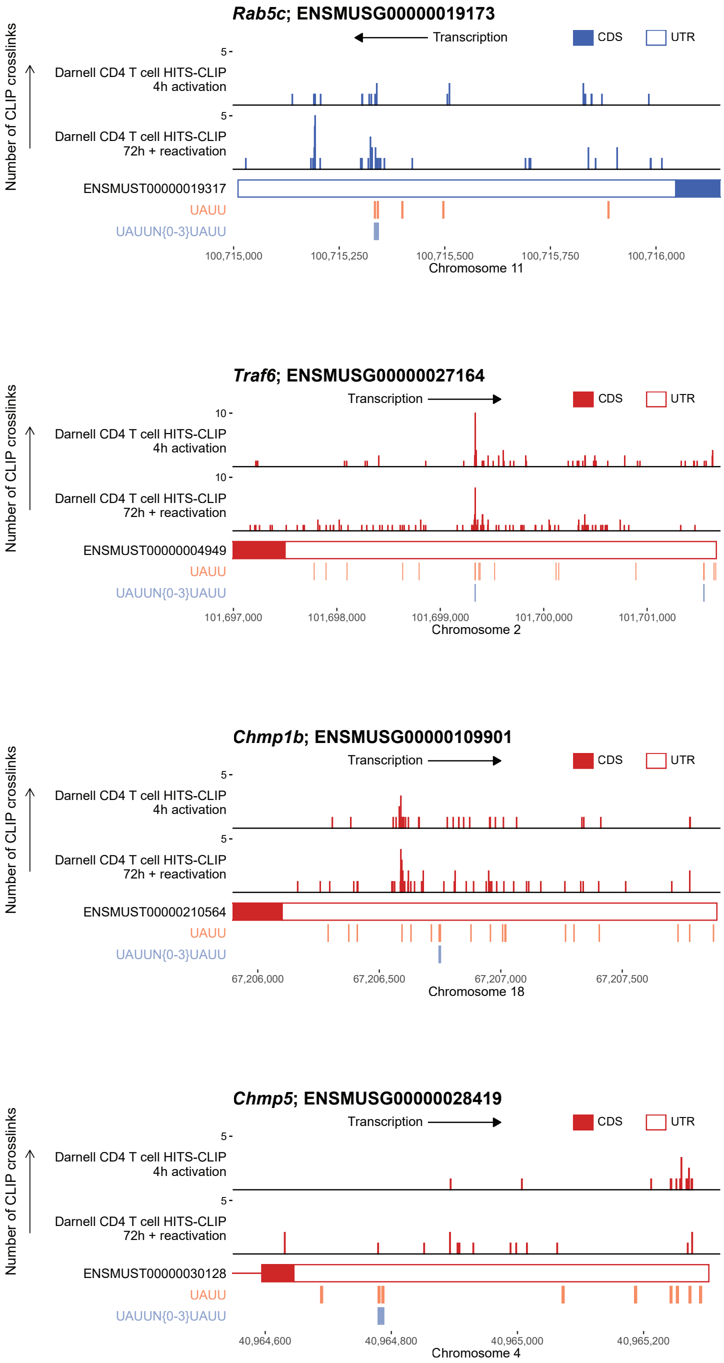

Supplementary Fig. 11 Direct and indirect mechanisms implicated in regulation of CTLA-4 turnover

Crosslinks over the indicated transcripts zoomed in on the 3'UTR in HITS-CLIP data. Occurrences of the UAUU motif are represented as vertical orange lines and 2 x UAUU separated by up to three nucleotides indicated as blue lines.

Supplementary Figure 12

a

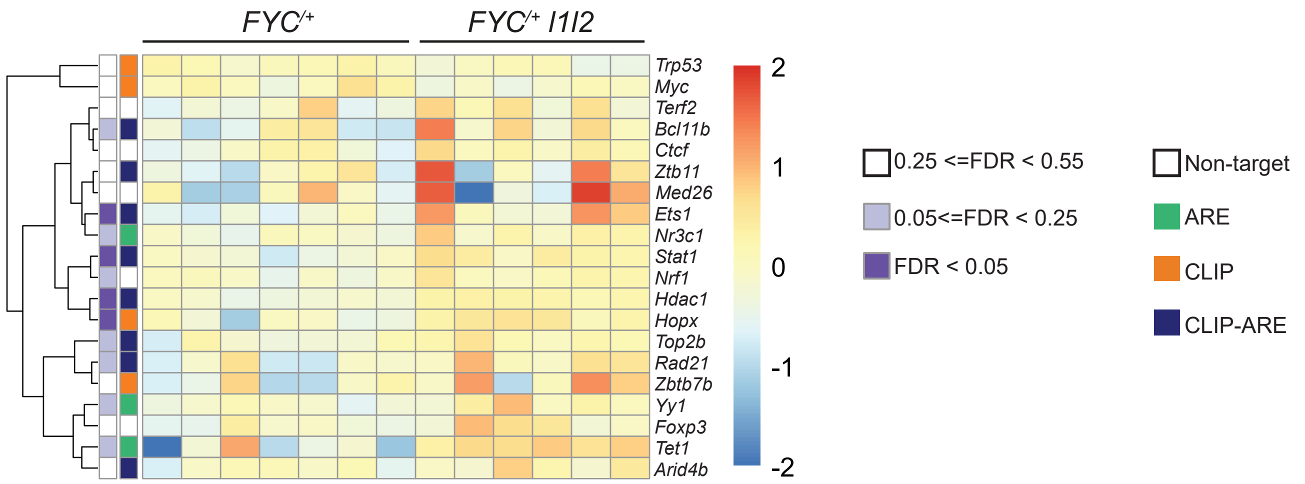

b

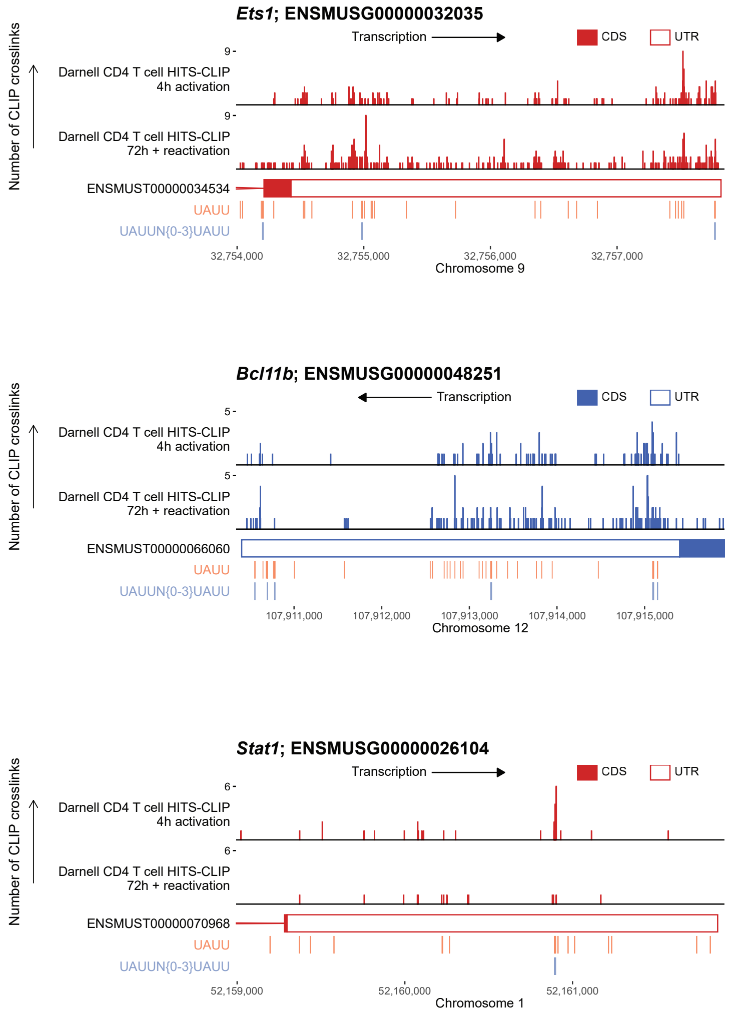

Supplementary Fig. 12 Transcription factors affecting endocytosis

a, Heatmap depicting transcriptional regulators that are frequently bound to genes within the endocytosis GSEA leading edge.

b, Crosslinks over the indicated transcripts zoomed in on the 3'UTR in HITS-CLIP data. Occurrences of the UAUU motif are represented as vertical orange lines and 2 x UAUU separated by up to three nucleotides indicated as blue lines.

Supplementary Figure 13

a

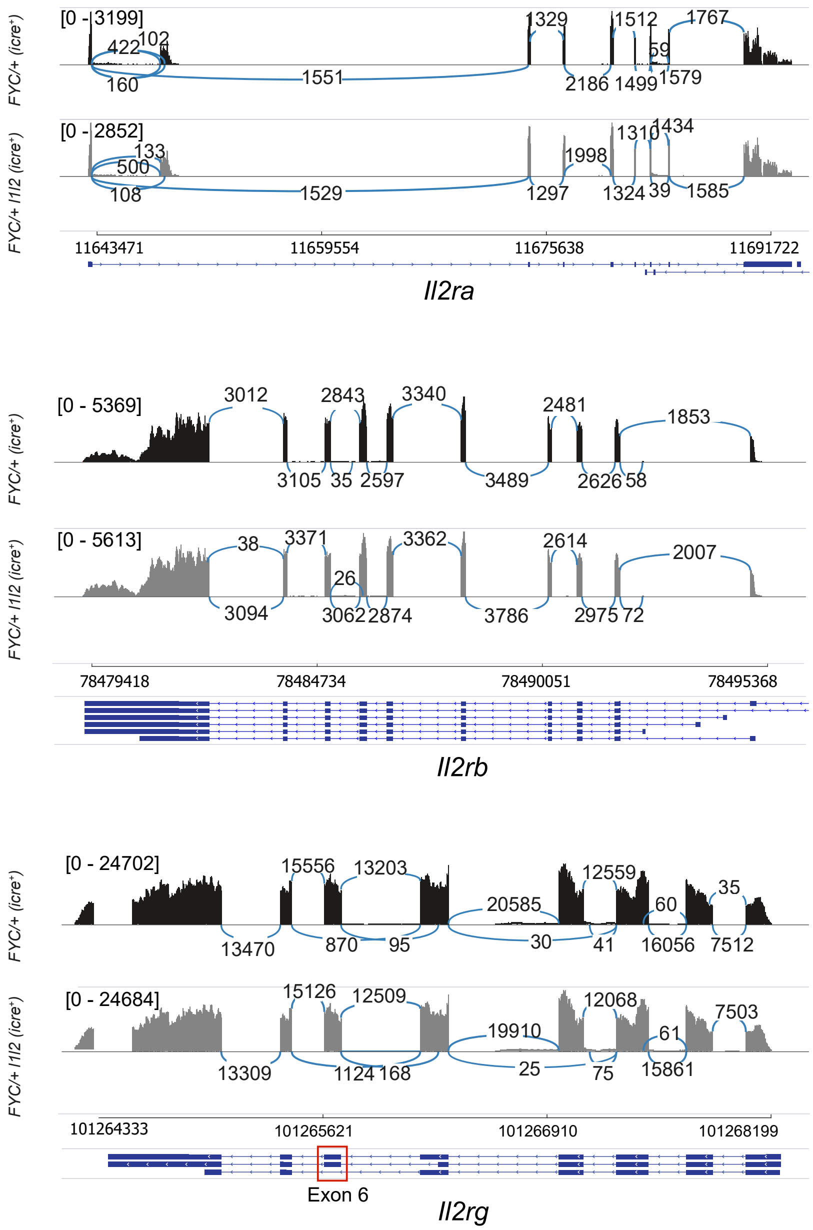

b

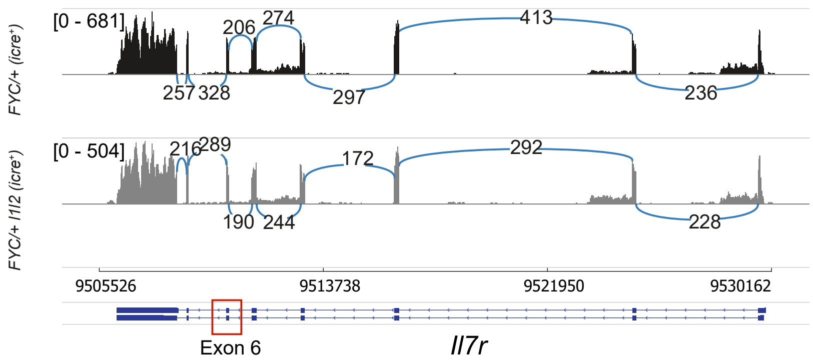

Supplementary Fig. 13 *Il2ra*, *Il2rb*, *Il2rg* and *Il7ra* transcript isoforms

a, Transcript isoforms for *Il2ra*, *Il2rb*, *Il2rg* showing read coverage and reads that map to exon–exon junctions (displayed in a Sashimi plot; arches: numbers indicate reads that map to that junction).

For *Il2rg*, exon 6 encoding the transmembrane domain is indicated by a red box.

b, Transcript isoforms for *Il7r*; exon 6 encoding the transmembrane domain is indicated by a red box.

Supplementary Figure 14

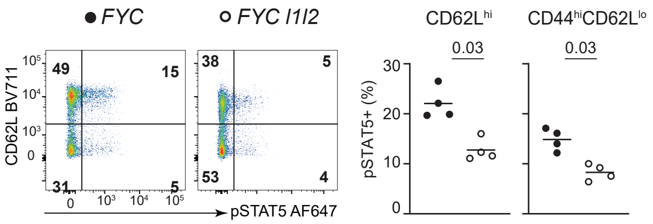

Supplementary Fig. 14 Diminished proportions of pSTAT5^{+} Treg *ex vivo* in *FYC I1I2* mice

Representative FACS plots of Treg from LN from male mice fixed directly *ex-viv*o and stained for pSTAT5; *FYC*, *FYC I*1I2 n=4, key as shown

Supplementary Figure 15

a

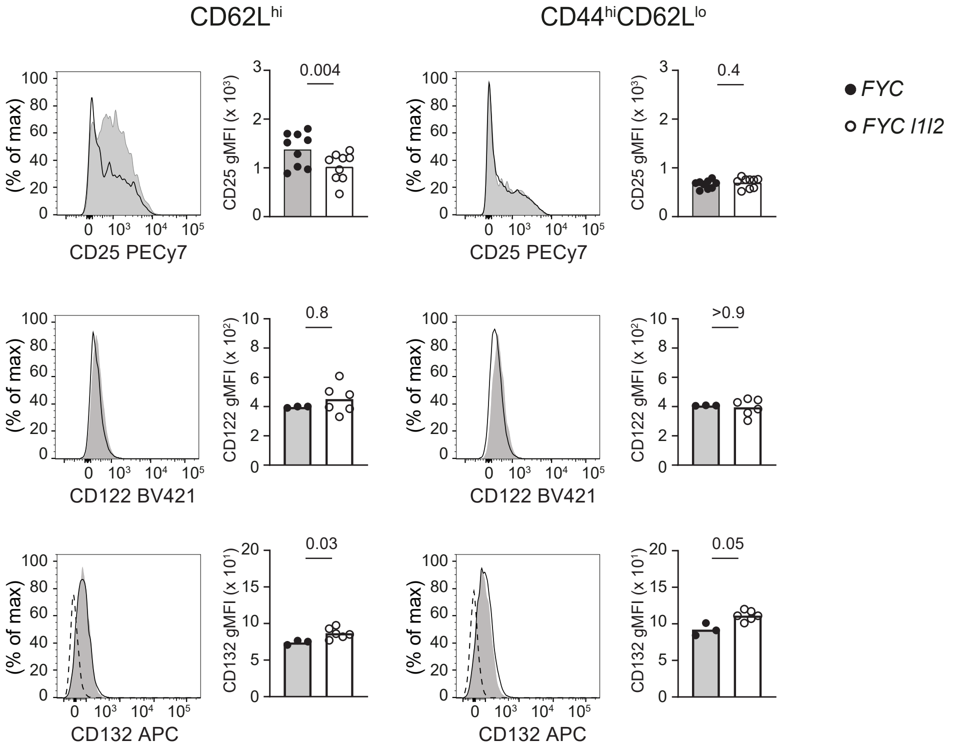

b

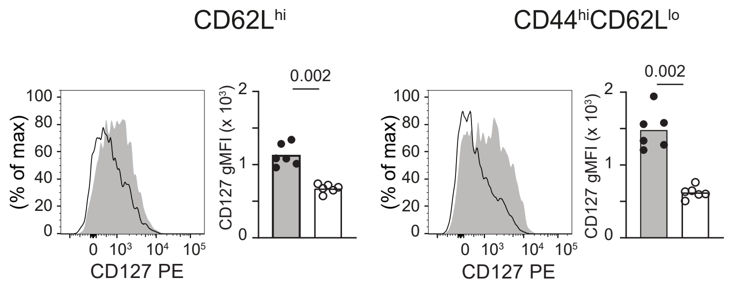

Supplementary Fig. 15 IL-2R and IL-7R expression in *FYC I1I2* male mice

a, Representative histogram overlays of CD25, CD122 and CD132 expression on nTreg (left panel) and eTreg (right panel) from spleen from *FYC* and *FYC I1I2* mice; gMFI of CD25, CD122 and CD132; the dashed line shown in the histogram overlays for CD132 represents the fluorescence minus one control; key as shown

b, Representative histogram overlays of CD127 expression on nTreg (left panel) and eTreg (right panel) from spleen from *FYC* and *FYC I1I2* mice and gMFI of CD127; key as in a. P values determined using Mann-Whitney.

Supplementary Figure 16

a

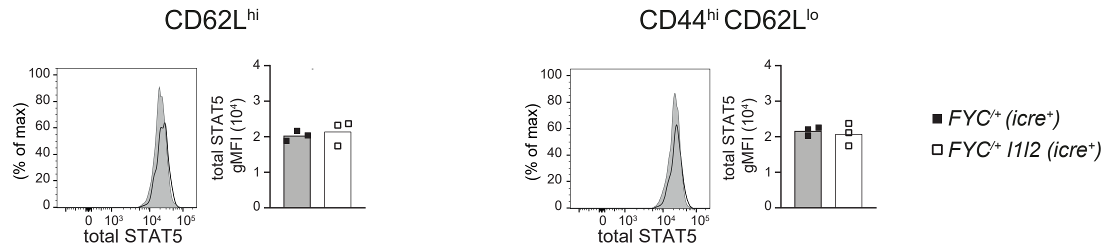

b

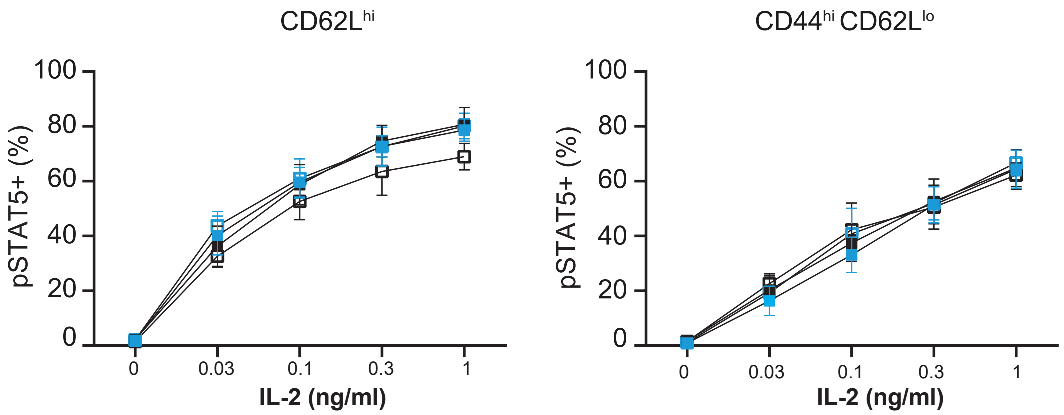

	■	□	□
■	0.9	0.7	<0.0001
■		0.7	<0.0001
□			<0.0001

■ *FYC/+ (icre-)*
■ *FYC/+ (icre+)*
□ *FYC/+ I1l2 (icre-)*
□ *FYC/+ I1l2 (icre+)*

c

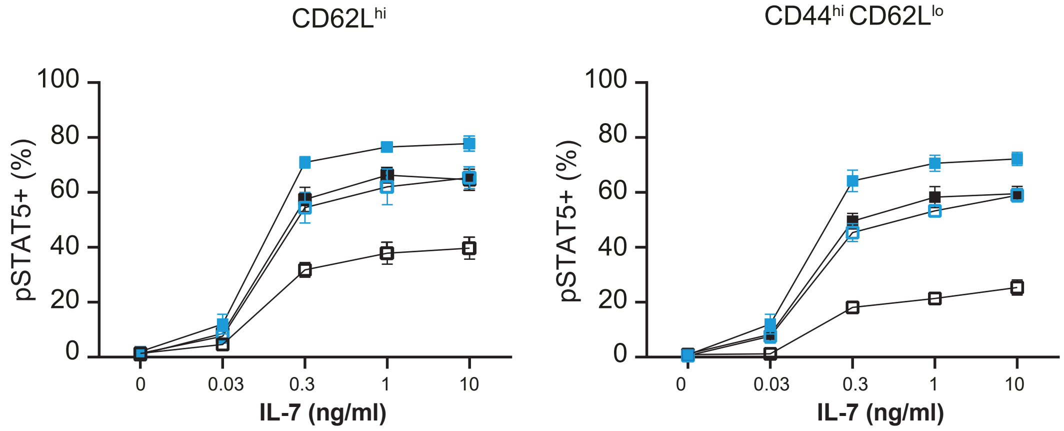

	■	□	□
■	<0.0001	<0.0001	<0.0001
■		0.5	<0.0001
□			<0.0001

	■	□	□
■	<0.0001	<0.0001	<0.0001
■		0.02	<0.0001
□			<0.0001

Supplementary Fig. 16 ZFP36L1 and ZFP36L2 promote Treg sensitivity to IL-2 and IL-7

a, Total STAT5 expression in YFP^{+} nTreg and eTreg; from *FYC*$^{/+}$ and *FYC*$^{/+}$ *l1l2* mice, n=3; key as shown

b, Frequency of pSTAT5^{+} cells detected in YFP^{+} (black symbol) and YFP^{-} (blue symbol) nTreg and eTreg from the spleen of female mice following stimulation for 30 minutes with a range of concentrations of IL-2; data presented as mean value ± sd, n= 7; key as shown

c, Frequency of pSTAT5^{+} cells detected in YFP^{+} and YFP^{-} nTreg and eTreg from female mice following stimulation for 30 minutes with a range of concentrations of IL-7; data presented as mean value ± sd, n=4; key as in b.

P values were determined using two-way ANOVA with multiple comparison, comparing the mean value between each genotype, and are represented in the table shown.

Supplementary Figure 17

a

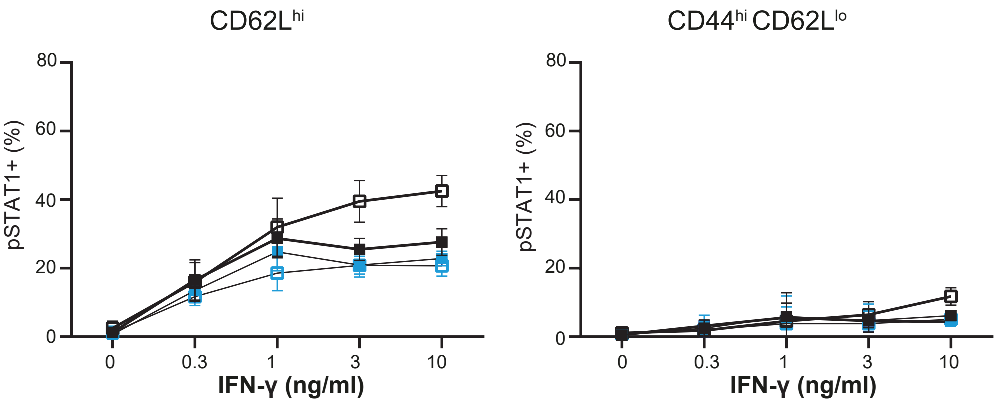

	■	□ (blue)	□
■ (blue)	0.006	0.2	<0.0001
■		<0.0001	<0.0001
□ (blue)			<0.0001

- ■ (blue) *FYC$^{/+}$ (icre^{-})*
- ■ *FYC$^{/+}$ (icre^{+})*
- □ (blue) *FYC$^{/+}$ I1I2 (icre^{-})*
- □ *FYC$^{/+}$ I1I2 (icre^{+})*

b

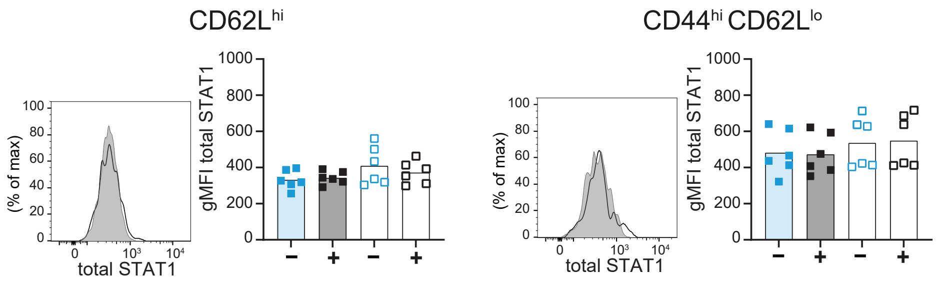

c

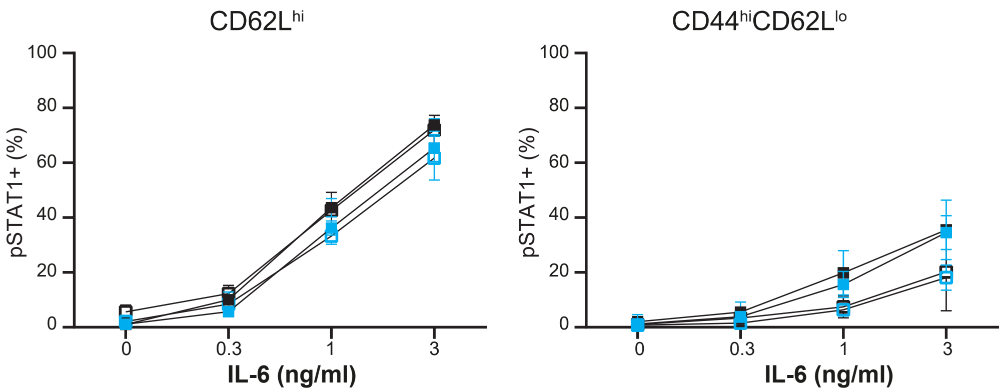

	■	□ (blue)	□
■ (blue)	0.006	0.9	0.0001
■		0.0004	0.8
□ (blue)			<0.0001

	■	□ (blue)	□
■ (blue)	0.7	0.001	0.01
■		<0.0001	0.0004
□ (blue)			0.8

Supplementary Fig. 17 ZFP36L1 and ZFP36L2 limit Treg sensitivity to IFNγ

a, Frequency of $pSTAT1^{+}$ cells detected in YFP^{+} and YFP^{-} nTreg and eTreg from female mice following stimulation for 30 minutes with a range of concentrations of IFNγ; data presented as mean value ± sd, n=6-11; key as shown

b, Total STAT1 expression in YFP^{+} and YFP^{-} nTreg and eTreg; key as in a

c, Frequency of $pSTAT1^{+}$ cells detected in YFP^{+} and YFP^{-} nTreg and eTreg from female mice following stimulation for 30 minutes with a range of concentrations of IL-6; data presented as mean value ± sd, n= 4-6; key as in a.

P values determined using two-way ANOVA with multiple comparison, comparing the mean value between each genotype, and are represented in the table shown (a, c).

Supplementary Figure 18

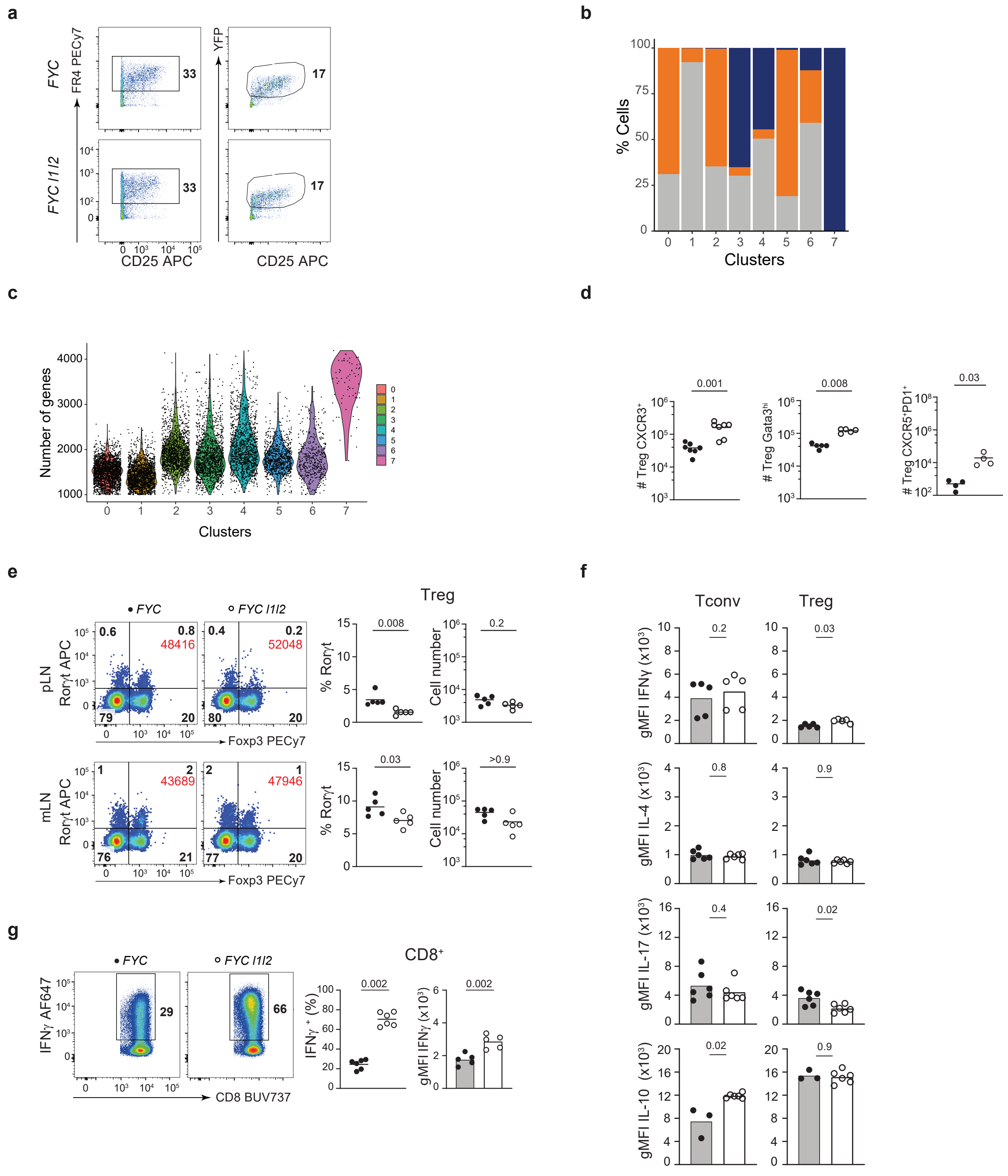

Supplementary Fig. 18 Activated phenotype of Treg from *FYC I1I2* mice

a, Gating strategy for sorting $CD4^+FR4^+CD25^+$ Treg cells for scRNA-seq

b, Percentage of cells identified as naïve (orange), effector (dark blue) or unassigned (grey) in each cluster;

c, Violin plot representing the number of genes detected in each cluster

d, Cell number for $CXCR3^+$, $GATA3^{hi}$ Treg and Tfr, in LN (for data in Fig. 7i, j, k); comparative values for *FYC I1I2* to *FYC*: 4-fold increase $CXCR3^+$, 3-fold increase in $GATA3^{hi}$, 50-fold increase in Tfr cell number

e, Representative flow cytometry plots (left panel) gated on $CD4^+$ cells; scatter plot (right panel) showing proportion of $ROR\gamma t^+$ Treg (as a % of all $FOXP3^+$ cells) and enumeration of $FOXP3^+ROR\gamma t^+$ Treg; upper panel – pLN, lower panel - mes LN; key as shown. The number of events acquired in the CD4 gate is shown in red.

f, gMFI for intracellular cytokine staining shown in Fig. 7l

g, IFNγ expression in CD8 cells. Splenocytes were stimulated with PMA/ionomycin for four hours in the presence of Brefeldin A, flow cytometry plots are gated on $CD8^+$ cells; n=6, key as shown. P values determined using Mann-Whitney test (d, e, f, g).

Supplementary Figure 19

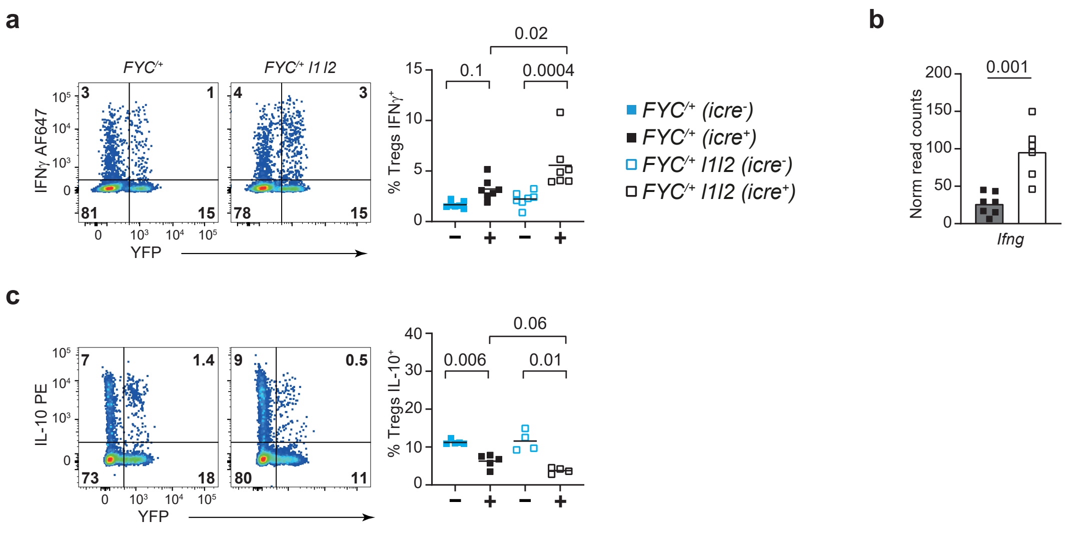

Supplementary Fig. 19 Increased production of IFNγ in *FYC*$^{/+}$ *I1I2* female mice is cell intrinsic

a, IFNγ expression in Tconv and Treg from *FYC*$^{/+}$ and *FYC*$^{/+}$ *I1I2* female mice. Splenocytes were stimulated with PMA/ionomycin for four hours in the presence of Brefeldin A; FACS plots gated on CD4$^+$ cells. Percentage values shown as a % of all CD4$^+$ YFP$^-$ cells (Tconv) or CD4$^+$YFP$^+$ cells (Treg). Key as shown.

b, Normalised read counts for *Ifng* in YFP$^+$ nTreg from *FYC*$^{/+}$ and *FYC*$^{/+}$ *I1I2* mice; p value determined using t test with FDR correction; key as in a.

c, IL-10 expression in Tconv and Treg from *FYC*$^{/+}$ and *FYC*$^{/+}$ *I1I2* female mice, cell stimulation as in a.

Supplementary Figure 20

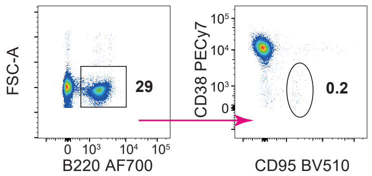

Supplementary Fig. 20 Gating strategy for GC B cells

Cells were pre-gated on live, single cells.

Supplementary Figure 21

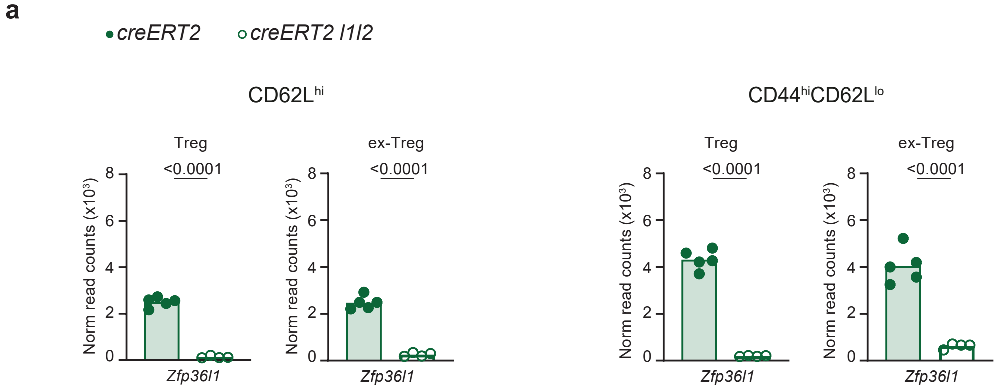

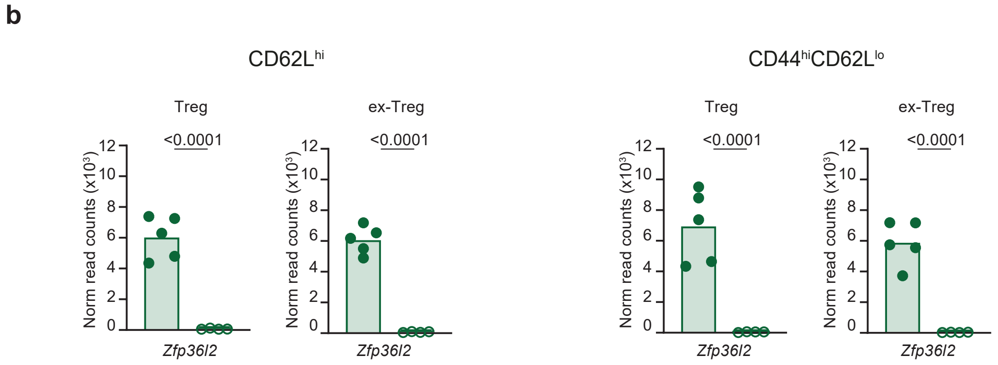

Supplementary Fig. 21

a, **b**, Normalised read counts across the loxP-flanked regions of the conditional alleles **a**, *Zfp36l1* and **b**, *Zfp36l2* within $CD4^+RFP^+GFP^+CD62L^{hi}$ (naïve Treg), $CD4^+RFP^+GFP^-CD62L^{hi}$ (naive ex-Treg), $CD4^+RFP^+GFP^+CD44^{hi}CD62L^{lo}$ (effector Treg), and $CD4^+RFP^+GFP^-CD44^{hi}CD62L^{lo}$ (effector ex-Treg); n=4-5; key as shown. Read counts were normalised using size factors derived from the overall DESeq2 analysis of all genes; p values determined using t-test with FDR correction.
