# Supplementary material for: ZFP36-family RNA-binding proteins in regulatory T cells reinforce immune homeostasis
Source: Nat Commun. 2025 May 6;16:4192. doi: 10.1038/s41467-025-58993-y (PMC12056042; doi:10.1038/s41467-025-58993-y)
Supplement: Supplementary file 2 — Description of Additional Supplementary Files [file 41467_2025_58993_MOESM2_ESM.pdf]

Description of Additional Supplementary Files:

Supplementary Data 1: Genes increased in expression comparing nTreg from *FYC*$^{/+}$ *l1l2* relative to *FYC*$^{/+}$ mice. Table only shows genes as differentially expressed if their FDR-adjusted p value was < 0.05. Analysis was performed using DESeq2 (v1.22.2; 10.1186/s13059-014-0550-8), using a two-sided Wald test and 'normal' log2 fold change shrinkage. CLIP targets and AU-rich elements are defined based on the transcript 3'UTRs.

Supplementary Data 2: Genes decreased in expression comparing nTreg from *FYC*$^{/+}$ *l1l2* relative to *FYC*$^{/+}$ mice. Table only shows genes as differentially expressed if their FDR-adjusted p value was < 0.05. Analysis was performed using DESeq2 (v1.22.2; 10.1186/s13059-014-0550-8), using a two-sided Wald test and 'normal' log2 fold change shrinkage. CLIP targets and AU-rich elements are defined based on the transcript 3'UTRs

Supplementary Data 3: ZFP36 family CLIP targets. A gene was designated a target if the 3'UTR contained a significant crosslink site (FDR < 0.05) with both antibodies in the iCLIP from data for ZFP36L1 in CD4$^+$ T cells activated for 24h with anti-CD3 and anti-CD28 obtained from GSE155087 or if an identical significant crosslink site in the 3'UTR was identified in at least two replicates for either of the HITS-CLIP datasets from GSE96074.

Supplementary Data 4: Gene sets used for GSEA. Hallmark, KEGG and GO gene sets were obtained from MSigDB {Liberzon.2015}, KEGG and Gene Ontology databases, respectively. TCR signalling pathway gene set was curated manually. Genes increased or decreased in Treg upon IL-2 or IL-7 treatment were identified using ImmGen common γ-chain cytokine RNA-seq data (GSE180020). For IFNγ treatment, microarray data of iTreg treated for 10h, compared with neutral conditions, was used (GSE38686). iCLIP targets are shown in orange, and genes with AU-rich elements in their 3'UTR in italics.

Supplementary Data 5: GSEA results. Gene set enrichment analysis was performed using the GSEA Preranked module of the GenePattern software package with default parameters except that 'collapse dataset' was set to 'No_collapse'. Genes were ranked based on -log10(p value) multiplied by the sign of the log2 fold change from the differential gene expression results for *FYC*$^{/+}$ *l1l2* relative to *FYC*$^{/+}$ nTreg. P values and adjusted p values are calculated using the in-built GSEA methods, based on comparison of actual enrichment scores with scores generated using 1000 random permutations of gene rankings.

Supplementary Data 6: Naive and effector Treg markers. Naïve and effector markers were defined as genes with FDR-adjusted p value < 0.0001, and log2-fold change in eTreg compared with nTreg < -1.5 or > 2, respectively from RNAseq data generated in house; p values and log2-fold change derived from DESeq2 analysis using a two-sided Wald test and 'normal' log2-fold change shrinkage.

Supplementary Data 7: Cluster markers. Table shows significantly enriched or decreased genes for the clusters 0-7 identified from single cell RNAseq data. To identify cluster-specific marker genes the FindMarkers function from Seurat was used with two-sided Wilcoxon Rank-Sum test, only considering genes detected in at least 25% of cells in at least one group for a given comparison.

Supplementary Data 8: IFNg signature genes. Human Reactome Interferon Gamma Signalling pathway genes were converted to mouse orthologues.

Supplementary Data 9: Antibodies used
