# Supplementary material for: ZFP36-family RNA-binding proteins in regulatory T cells reinforce immune homeostasis
Source: Nat Commun. 2025 May 6;16:4192. doi: 10.1038/s41467-025-58993-y (PMC12056042; doi:10.1038/s41467-025-58993-y)
Supplement: Supplementary file 4 — Reporting Summary [file 41467_2025_58993_MOESM4_ESM.pdf]

nature portfolio

Corresponding author(s): Martin Turner

Last updated by author(s): Mar 17, 2025

Reporting Summary

Nature Portfolio wishes to improve the reproducibility of the work that we publish. This form provides structure for consistency and transparency in reporting. For further information on Nature Portfolio policies, see our Editorial Policies and the Editorial Policy Checklist.

Statistics

For all statistical analyses, confirm that the following items are present in the figure legend, table legend, main text, or Methods section.

n/a	Confirmed	
☐	☒	The exact sample size (*n*) for each experimental group/condition, given as a discrete number and unit of measurement
☐	☒	A statement on whether measurements were taken from distinct samples or whether the same sample was measured repeatedly
☐	☒	The statistical test(s) used AND whether they are one- or two-sided<br>*Only common tests should be described solely by name; describe more complex techniques in the Methods section.*
☒	☐	A description of all covariates tested
☐	☒	A description of any assumptions or corrections, such as tests of normality and adjustment for multiple comparisons
☐	☒	A full description of the statistical parameters including central tendency (e.g. means) or other basic estimates (e.g. regression coefficient) AND variation (e.g. standard deviation) or associated estimates of uncertainty (e.g. confidence intervals)
☐	☒	For null hypothesis testing, the test statistic (e.g. *F*, *t*, *r*) with confidence intervals, effect sizes, degrees of freedom and *P* value noted<br>*Give P values as exact values whenever suitable.*
☒	☐	For Bayesian analysis, information on the choice of priors and Markov chain Monte Carlo settings
☒	☐	For hierarchical and complex designs, identification of the appropriate level for tests and full reporting of outcomes
☒	☐	Estimates of effect sizes (e.g. Cohen's *d*, Pearson's *r*), indicating how they were calculated

Our web collection on statistics for biologists contains articles on many of the points above.

Software and code

Policy information about availability of computer code

Data collection	Publicly available datasets were downloaded from GEO (https://www.ncbi.nlm.nih.gov/geo/).
Data analysis	Details are described in the Methods section. RNA-seq data were trimmed using Trim Galore v 0.6.5, and mapped to the GRCm38 mouse genome using Hisat2 v2.1.0. BAM files were imported into Seqmonk (v1.47.0 http://www.bioinformatics.babraham.ac.uk/projects/seqmonk/) excluding those with mapping quality < 30, and reads were quantified over merged mRNA isoforms from the GRCm38 v90 annotation, using the RNA-seq quantitation pipeline. Downstream analysis was performed in R (v4.1.2). Differential expression analysis comparing cKO with control samples was performed using DESeq2 (v1.22.2). Gene set enrichment analysis was performed using the GSEAPreranked module (v7.4.0) of the GenePattern software package (v3.9_080823_b401; as indicated in the Methods). Custom gene sets were uploaded for the analysis: the Hallmark apoptosis gene set was obtained from MSigDB (v6.2); and gene names converted to mouse orthologues using biomaRt whilst the TCR signaling pathway gene set was curated manually (see Table S4).<br>For sc RNA seq fastq files were first split based on whether an exact match to one of the hashtag oligos for multiplexing was found starting in position 11 in the "R2" feature barcoding fastq file. The separated multiplexing and antibody-derived tag fastq files, together with the gene expression fastq files, were then processed using Cell Ranger v6.1.2, first with cellranger multi, using the GRCm38 mouse genome reference, followed by aggregation of the control and knockout data from the per sample outputs, using cellranger aggr. Further analysis was performed using the Seurat package (v4.1.0) in R (v4.1.2). Cells containing below 1500 and over 14000 molecule counts for either hashtag 1 or hashtag 2 oligo conjugated antibodies were removed to filter out putative empty droplets, or doublets respectively. Additionally, cells containing over 5.5 % mitochondria-derived gene expression reads, over 10% reads originating from a single gene, or in which fewer than 1000 or more than 4200 genes were detected were removed. After filtering the top 500 variable genes were identified, and these were scaled and used as input for principal component analysis. The top 15 principal components were then used as input for Seurat's graph-based clustering approach (FindNeighbors followed by FindClusters functions; resolution 0.5; all other parameters default). These 15 principal components were also used as input to RunUMAP for further dimensionality reduction and data visualization. To identify cluster-specific marker genes the

FindMarkers function was used. The SCINA package (v1.2.0) was used to assign cell type identities based on previous knowledge related to naïve/effector cells (using the gene list in Table S6). For iCLIP data all data was analyzed using the iCount pipeline (v2.0.1.dev) on the Genialis platform (now obsolete; iCount now hosted on https://app.flow.bio/).
Heatmaps were plotted using the pheatmap R package, with a threshold set on the fill color such that values above/below the maximum/minimum threshold were assigned the maximum/minimum colors in the scale.
To identify transcriptional regulators of endocytosis genes, ReMap (2022 release) DNA binding peaks {Hammal.2021} overlapping with 1 kb windows flanking the transcription start sites of all genes within the GSEA leading edge were downloaded from the UCSC genome browser.
CLIP crosslinks over the 3'UTRs of selected transcripts were visualized using a shiny app (https://github.com/LouiseMatheson/iCLIP_visualisation_shiny), without filtering for significant sites.
The R code underlying the RNA-seq and iCLIP analysis and visualization has been deposited in GitHub, and released with Zenodo under the DOIs 10.5281/zenodo.15021467 [https://doi.org/10.5281/zenodo.15021467] and 10.5281/zenodo.14982514 [https://doi.org/10.5281/zenodo.14982514].

For manuscripts utilizing custom algorithms or software that are central to the research but not yet described in published literature, software must be made available to editors and reviewers. We strongly encourage code deposition in a community repository (e.g. GitHub). See the Nature Portfolio guidelines for submitting code & software for further information.

Data

Policy information about availability of data

All manuscripts must include a data availability statement. This statement should provide the following information, where applicable:

- Accession codes, unique identifiers, or web links for publicly available datasets
- A description of any restrictions on data availability
- For clinical datasets or third party data, please ensure that the statement adheres to our policy

All data generated and analysed during this study are included in the manuscript and supplementary information or have been made available in public repositories as follows:
Sequencing data from RNA seq experiments performed are publicly available from the Gene Expression Omnibus (GEO) database (https://www.ncbi.nlm.nih.gov/geo/) under accession code GSE244621;
Previously published datasets are available on GEO under the accessions
GSE180020; GSE38686; GSE96074; GSE155087

Research involving human participants, their data, or biological material

Policy information about studies with human participants or human data. See also policy information about sex, gender (identity/presentation), and sexual orientation and race, ethnicity and racism.

Reporting on sex and gender	*Use the terms sex (biological attribute) and gender (shaped by social and cultural circumstances) carefully in order to avoid confusing both terms. Indicate if findings apply to only one sex or gender; describe whether sex and gender were considered in study design; whether sex and/or gender was determined based on self-reporting or assigned and methods used. Provide in the source data disaggregated sex and gender data, where this information has been collected, and if consent has been obtained for sharing of individual-level data; provide overall numbers in this Reporting Summary. Please state if this information has not been collected. Report sex- and gender-based analyses where performed, justify reasons for lack of sex- and gender-based analysis.*
Reporting on race, ethnicity, or other socially relevant groupings	*Please specify the socially constructed or socially relevant categorization variable(s) used in your manuscript and explain why they were used. Please note that such variables should not be used as proxies for other socially constructed/relevant variables (for example, race or ethnicity should not be used as a proxy for socioeconomic status). Provide clear definitions of the relevant terms used, how they were provided (by the participants/respondents, the researchers, or third parties), and the method(s) used to classify people into the different categories (e.g. self-report, census or administrative data, social media data, etc.) Please provide details about how you controlled for confounding variables in your analyses.*
Population characteristics	*Describe the covariate-relevant population characteristics of the human research participants (e.g. age, genotypic information, past and current diagnosis and treatment categories). If you filled out the behavioural & social sciences study design questions and have nothing to add here, write "See above."*
Recruitment	*Describe how participants were recruited. Outline any potential self-selection bias or other biases that may be present and how these are likely to impact results.*
Ethics oversight	*Identify the organization(s) that approved the study protocol.*

Note that full information on the approval of the study protocol must also be provided in the manuscript.

Field-specific reporting

Please select the one below that is the best fit for your research. If you are not sure, read the appropriate sections before making your selection.

[x] Life sciences [ ] Behavioural & social sciences [ ] Ecological, evolutionary & environmental sciences

Life sciences study design

All studies must disclose on these points even when the disclosure is negative.

Sample size	Sample sizes have not been determined in advance due to the novelty of this research and with no prior knowledge of the effect size of the genotype. Sample sizes were influenced by animal availability and experiments were repeated to enable statistical verification.
Data exclusions	Data was not excluded
Replication	Experiments have been repeated on at least two independent occasions and data has shown reproducibility.
Randomization	Mice were not randomised but age and sex matched as appropriate.<br>Tissue collection, and analysis of samples was performed and acquired in a randomized fashion.
Blinding	Investigators were not blinded to group allocation or sample collection since the experiments were all designed and performed by the same investigators.

Reporting for specific materials, systems and methods

We require information from authors about some types of materials, experimental systems and methods used in many studies. Here, indicate whether each material, system or method listed is relevant to your study. If you are not sure if a list item applies to your research, read the appropriate section before selecting a response.

Materials & experimental systems

n/a	Involved in the study	
☐	☒	Antibodies
☒	☐	Eukaryotic cell lines
☒	☐	Palaeontology and archaeology
☐	☒	Animals and other organisms
☒	☐	Clinical data
☒	☐	Dual use research of concern
☒	☐	Plants

Methods

n/a	Involved in the study	
☒	☐	ChIP-seq
☐	☒	Flow cytometry
☒	☐	MRI-based neuroimaging

Antibodies

Antibodies used

Antibodies Conjugate Source Identifier clone Dilution
B220 AF700 BioLegend 103232 RA3-6B2 1 in 200
B220 Biotin Invitrogen 13-0452-85 RA3-6B2 1 in 200
B220 BUV395 BD Biosciences 563793 RA3-6B2 1 in 400
CD11b PerCPCy5.5 BioLegend 101227 M1/70 1 in 200
CD11c BV605 BioLegend 117333 N418 1 in 200
CD16/32 unconjugated BioXcell BE0008 2.4G2 1 in 2000
CD122 BV421 BD Biosciences 562960 Tm-b1 1 in 100
CD122 PE BD Biosciences 553362 Tm-b1 1 in 100
CD127 PE BioLegend 135010 A7R34 1 in 100
CD132 APC BioLegend 132307 TUGm2 1 in 200
CTLA-4 (CD152) APC BD Biosciences 564331 UC10-4F10-11 1 in 200
CTLA-4 (CD152) BV605 BioLegend 106323 UC10-4B9 1 in 100
CD172a PECy7 BioLegend 144007 P84 1 in 200
CD24 BV510 BD Biosciences 563115 M1/69 1 in 200
CD25 PECy7 eBioscience 25-0251-81 PC61.5 1 in 200
CD25 APC BioLegend 102012 PC61 1 in 400
CD25 BB515 BD Biosciences 564424 PC61 1 in 200
CD25 PerCPCy5.5 eBioscience 45-0251-82 PC61.5 1 in 200
ICOS (CD278) BV421 BioLegend 313524 C398.4A 1 in 200
CD279 (PD1) APC eBioscience 17-9985-82 J43 1 in 100
CD3 FITC Tonbo 35-0031-U100 145-2C11 1 in 400
CD38 PECy7 BioLegend 102718 90 1 in 200
CD4 BUV395 BD Biosciences 563790 GK1.5 1 in 400
CD44 BV785 BioLegend 103041 IM7 1 in 800
CD44 PE BioLegend 12-0441-82 IM7 1 in 1000
CD5 APC BD Biosciences 561895 53-7.3 1 in 800

CD62L PE BioLegend 104407 MEL-14 1 in 800
CD62L AF647 BioLegend 104421 MEL-14 1 in 400
CD62L BV421 BioLegend 104435 MEL-14 1 in 400
CD62L BV711 BioLegend 104445 MEL-14 1 in 800
CD64 FITC BioLegend 139315 X54-5/7.1 1 in 400
CD69 APC BioLegend 104504 H1.2F3 1 in 200
CD8a BUV737 BD Biosciences 564297 53-6.7 1 in 200
CD8a BV605 BioLegend 100744 53-6.7 1 in 200
CD8a BV711 BioLegend 100747 53-6.7 1 in 200
CD8a Biotin BioLegend 100704 53-6.7 1 in 200
CD80 APC BioLegend 104713 16-10A1 1 in 200
CD86 BV785 BioLegend 105043 GL-1 1 in 200
CD95 BV510 BD Biosciences 563646 Jo2 1 in 100
CXCR3 PEDazz594 BioLegend 155914 S18001A 1 in 200
CXCR5 PEDazz594 BioLegend L138D7 L138D7 1 in 100
Foxp3 eF450 eBioscience 48-5773-80 FJK-16s 1 in 100
Foxp3 PECy7 eBioscience 25-5773-80 FJK-16s 1 in 100
F4/80 Biotin BioLegend 123106 BM8 1 in 200
F4/80 FITC BioLegend 123107 BM8 1 in 400
FR4 PECy7 Thermofisher 25-5445-80 12A5 1 in 400
GFP AF488 Biolegend 338008 FM264G 1 in 200
MHCII BUV737 BD Biosciences 748845 M5/114.15.2 1 in 800
TCRb BUV737 BD Biosciences 564799 H57-597 1 in 200
TCRb PE Cy7 BioLegend 109222 H57-597 1 in 400
XCR1 PE BioLegend 148204 ZET 1 in 200

GATA3 BV421 BD Biosciences 563349 L50-823 1 in 200
Helios (IKZF2) PE BioLegend 137216 22F6 1 in 200
IL-4 BV711 BD Biosciences 564005 11B11 1 in 200
IL-10 PE BioLegend 505008 JES5-16E3 1 in 200
IL-17a PE Cy7 eBioscience 25-7177-82 eBio17B7 1 in 200
IFNγ AF647 BD Biosciences 557735 XMG1.2 1 in 200
TNF BV650 BioLegend 506333 MP6-XT22 1 in 200
NOTCH1 PE BioLegend 629105 mN1A 1 in 100
Nur77 PE Thermofisher 12-5965 12.14 1 in 100
RFP unconjugated Rockland 600-401-379 Rabbit polyclonal 1 in 2000
RORgt APC eBioscience 17-6981-80 B2D 1 in 200
STAT1 AF647 BD Biosciences 558560 Clone 1/Stat1 1 in 200
STAT1(Y701) AF647 BD Biosciences 612597 4a 1 in 200
STAT5alpha/beta unconjugated Thermofisher MA5-32452 JJ08-78 1 in 200
STAT5a (Y694) AF647 BD Biosciences 612599 47/Stat5(pY694) 1 in 200
ZFP36L1 Unconjugated Cell Signaling Technology 30894 Rabbit monoclonal 1 in 200
anti-Rabbit IgG AF647 Jackson Immunoresearch 711-605-152 Donkey polyclonal 1 in 2000
Streptavidin BV421 BD Biosciences 563259 1 in 600

IgG2c Unconjugated Southern Biotech 1079-01 goat polyclonal 1 in 1000
IgG2c biotin Southern Biotech 1079-08 goat polyclonal 1 in 5000
IgE Unconjugated BD Biosciences 553413 R35-72 1 in 250
IgE biotin BD Biosciences 553419 R35-118 1 in 500
Streptavidin HRP BD Biosciences 555428 1 in 250
Streptavidin HRP Southern Biotech 7100-05 1 in 8000

Totalseq oligo-conjugated antibodies
TotalSeq™-B0301 anti-mouse Hashtag 1 Antibody BioLegend 155831 M1/42 ; 30-F11 1 in 200
TotalSeq™-B0302 anti-mouse Hashtag 2 Antibody BioLegend 155833 M1/42 ; 30-F11 1 in 200
TotalSeq™-B0198 anti-mouse CD127 (IL-7Rα) Antibody BioLegend 135055 A7R34 1 in 250

Validation

We have validated the antibody for ZFP36L1 using CrispR mediated deletion in T cells.
For all other primary antibodies we have relied on validation provided by the supplier.

Animals and other research organisms

Policy information about studies involving animals; ARRIVE guidelines recommended for reporting animal research, and Sex and Gender in Research

Laboratory animals	Mice (Mus musculus) were maintained on a C57BL/6J background and used between 7-20 weeks of age
Wild animals	None were used
Reporting on sex	Both male and female mice were used in this study. As Foxp3 is on the X chromosome, sex informed study design to compare control and conditional knockout animals as indicated in the text and figure legends.
Field-collected samples	No field samples were collected
Ethics oversight	Babraham Institute Animal Welfare and Ethical Review body and United Kingdom Home Office legislation

Note that full information on the approval of the study protocol must also be provided in the manuscript.

Plants

Seed stocks	*Report on the source of all seed stocks or other plant material used. If applicable, state the seed stock centre and catalogue number. If plant specimens were collected from the field, describe the collection location, date and sampling procedures.*
Novel plant genotypes	*Describe the methods by which all novel plant genotypes were produced. This includes those generated by transgenic approaches, gene editing, chemical/radiation-based mutagenesis and hybridization. For transgenic lines, describe the transformation method, the number of independent lines analyzed and the generation upon which experiments were performed. For gene-edited lines, describe the editor used, the endogenous sequence targeted for editing, the targeting guide RNA sequence (if applicable) and how the editor was applied.*
Authentication	*Describe any authentication procedures for each seed stock used or novel genotype generated. Describe any experiments used to assess the effect of a mutation and, where applicable, how potential secondary effects (e.g. second site T-DNA insertions, mosiacism, off-target gene editing) were examined.*

Flow Cytometry

Plots

Confirm that:

- [x] The axis labels state the marker and fluorochrome used (e.g. CD4-FITC).
- [x] The axis scales are clearly visible. Include numbers along axes only for bottom left plot of group (a 'group' is an analysis of identical markers).
- [x] All plots are contour plots with outliers or pseudocolor plots.
- [x] A numerical value for number of cells or percentage (with statistics) is provided.

Methodology

Sample preparation	Single cell suspensions were isolated from the tissue of interest and prepared by mechanical dissociation into RPMI medium containing 2% FCS and 10mM HEPES and filtered (40um) and kept cold or on ice where appropriate. For analysis directly ex vivo, LN cell suspensions were prepared directly into fixation buffer as indicated in the Methods.
Instrument	BD Fortessa flow cytometer
Software	FACSDiva and Flowjo V10.8
Cell population abundance	Cell populations were sorted to >95% purity, confirmed by flow analysis, and were counted to confirm cell numbers
Gating strategy	Dead cells were excluded using fixable viability dye, and forward and side scatter properties were used to define lymphocytes and exclude doublets.

- [x] Tick this box to confirm that a figure exemplifying the gating strategy is provided in the Supplementary Information.
